# Prevalence of copper-resistant *Pseudomonas syringae* pv. *actinidiae* biovar 3 in Jiangxi Province, China

**DOI:** 10.3389/fmicb.2026.1824303

**Published:** 2026-04-29

**Authors:** Mingfeng Yan, Zhenrui He, Xinshen Li, Shuijin Huang

**Affiliations:** 1 Institute of Plant Protection, Jiangxi Academy of Agricultural Sciences, Nanchang, China; 2 Jiangxi Provincial Key Laboratory of Agricultural Non-point Source Pollution Control and Waste Comprehensive Utilization, Jiangxi Academy of Agricultural Sciences, Nanchang, China

**Keywords:** bacterial canker, biovar 3, copper sensitivity, kiwifruit, *Pseudomonas syringae* pv. *actinidiae*

## Abstract

Bacterial canker of the kiwifruit caused by the etiological agent *Pseudomonas syringae* pv. *actinidiae* (Psa) is the most severe disease in kiwifruit production. Since 2008, a hypervirulent Psa biovar 3 has spread rapidly worldwide. This study presents the first systematic characterization of Psa in Jiangxi Province, a major kiwifruit-growing region of China. Among 42 bacterial isolates collected from six production areas, all were identified as the highly pathogenic Psa biovar 3 through morphological, PCR-based, and multilocus sequence analyses. *In vitro* bioassays revealed that tetramycin exhibited the strongest inhibitory activity against a representative strain (F7), with an EC_50_ of approximately 0.002 mg/L, followed by bronopol, ethylicin, and benziothiazolinone. Notably, no copper-sensitive strains were detected, and all isolates showed a high level of copper resistance, with minimum inhibitory concentrations (MICs) values of 1.80–2.60 mM of copper sulfate, far above the conventional resistance threshold. These results demonstrate that Psa biovar 3 is the dominant pathogen in Jiangxi and that local populations pose a significant risk of copper resistance, highlighting the urgent need for integrated management strategies that reduce reliance on copper-based bactericides.

## 1 Introduction

Kiwifruit (*Actinidia chinensis* Planch.), a perennial vine native to China, bears fruits of high nutritional and medicinal value (Vanneste, 2017). Since its introduction to New Zealand in the last century, it has been widely cultivated in many regions worldwide (Vanneste, 2017). With growing consumer demand for its taste and health benefits, the cultivation area and yield of kiwifruit have increased annually, accompanied by a rise in disease risks. Among these, bacterial canker caused by *Pseudomonas syringae* pv. *actinidiae* (Psa) is one of the most severe threats to global kiwifruit production (Donati et al., 2020; Figueira et al., 2020). First reported in Japan in 1984 (Takikawa et al., 1989), the disease has since spread rapidly to major kiwifruit-growing countries, including China, the United States, Portugal, Italy, New Zealand, Chile, Spain, Korea, Turkey, and France (Abelleira et al., 2011; Vanneste et al., 2011; Bastas and Karakaya, 2012; Butler et al., 2013; Vanneste et al., 2014). Its worldwide dissemination has led to substantial economic losses, posing a serious challenge to sustainable kiwifruit cultivation.

Psa is not a homogeneous pathovar (Vanneste, 2017). Based on genetic diversity, phytotoxin production, and pathogenicity, global Psa populations have been classified into six biovars (biovars 1–6). Biovar 1, first identified in Japan in 1984 and later detected in Italy in 1992, produces phaseolotoxin (Fujikawa et al., 2020). Biovar 2, reported only in Korea since 1988, produces coronatine but not phaseolotoxin (Kim et al., 2017). Biovar 3, currently the most widespread and pathogenic group, does not produce phaseolotoxin or coronatine but carries type III effector genes such as *hopH1* and *hopZ5* (Zhao et al., 2019; Pei et al., 2023). Biovar 4, identified in New Zealand, Australia, and France, is a low-virulence group causing only leaf symptoms without notable stem lesions and has been designated as *P. syringae* pv. *actinidifoliorum* (Psaf) (Cunty et al., 2016). Biovar 5, emerging in Japan in 2012 and restricted to certain regions, lacks both the coronatine biosynthesis gene cluster (which is conserved in biovar 2) and the phaseolotoxin biosynthesis gene cluster (which is conserved in biovar 1) (Poulter et al., 2017). Finally, Biovar 6, discovered in Nagano, Japan, in 2015, is capable of producing both phaseolotoxin and coronatine (Fujikawa and Sawada, 2019).

In China, bacterial canker was first observed in Hunan Province in 1985 and has since spread to major kiwifruit-growing regions, including Sichuan, Shaanxi, Shanxi, Anhui, Fujian, and Hubei (Butler et al., 2013; He et al., 2019; Pei et al., 2023). Although several studies have reported the occurrence and identification of the pathogen in different provinces, the number of Psa strains collected and characterized in China remains limited. Consequently, the genetic diversity and biovar composition of *P. syringae* pv. *actinidiae* populations across different geographical regions in China remain poorly understood. Clarifying the genotypes, pathogenicity, and regional prevalence of Psa populations is essential for developing effective control strategies and for elucidating pathogenic mechanisms and key virulence determinants.

Recent studies indicated that Psa strains from Shaanxi Province in northwestern China and Sichuan Province in southwestern China belong to biovar 3 (He et al., 2019; Pei et al., 2023). However, the biovar status of Psa populations in Jiangxi Province in southeastern China remains unclear. Therefore, this study aims to isolate and characterize Psa strains from kiwifruit in Jiangxi Province, China, with confirmatory identification at the pathogen level. Furthermore, biovar-specific primers and multilocus sequence analysis (MLSA) were employed to clarify the genetic relationships among *P. syringae* pv. *actinidiae* strains from different geographical regions in Jiangxi Province. Finally, fungicides effective against these strains were screened, and copper resistance in Psa isolates from Jiangxi was monitored for the first time. These findings provide a theoretical foundation for the scientific prevention and management of kiwifruit bacterial canker.

## 2 Materials and methods

### 2.1 Bacterial isolates

Between January 2023 and April 2024, a collection of 42 Psa strains was isolated and identified from over 120 samples collected across six major kiwifruit-producing areas of Jiangxi Province, China: Fengxin county (28.72° N, 115.16° E), Gaoan county (28.44° N, 115.36° E), Jingdezhen county (29.29° N, 117.20° E), Wuning county (29.28° N, 115.05° E), Shangrao county (28.46° N, 117.94° E), and Xunwu county (24.57° N, 115.38° E).

These strains were isolated from diseased leaves, trunks or branches of kiwifruit, and from three kiwifruit species (Hongyang, Donghong, and Hort l6A) (Table 1). Pieces of symptomatic tissue samples were cut with a sterile blade and surface sterilized in 10% ethanol solution for 30s. Small pieces were aseptically removed from the margin of the infected tissues, and ground and inoculated on NA medium agar plate and incubated at 28 °C for 24–48 h in the dark (Mazzaglia et al., 2011). The single colonies were picked and restreaked on the same medium. Finally, the colonies of bacterial isolates were placed into sterile distilled water containing 15% glycerol and kept at −80 °C.

**Table 1:** *Pseudomonas syringae* pv. actinidiae isolates obtained from the kiwifruit samples in this study.

Number	Strain name	Region	Cultivar	Tissue	Year	Psa biovar	MIC to CuSO4 (mM)
1	F1	Fengxin, Jiangxi, China	Hongyang	Branch	2023	3	2.4
2	F2	Fengxin, Jiangxi, China	Hongyang	Branch	2023	3	1.8
3	F3	Fengxin, Jiangxi, China	Hongyang	Trunk	2023	3	2.0
4	F4	Fengxin, Jiangxi, China	Donghong	Branch	2023	3	2.4
5	F5	Fengxin, Jiangxi, China	Donghong	Branch	2023	3	2.4
6	F6	Fengxin, Jiangxi, China	Donghong	Leaf	2023	3	2.4
7	F7	Fengxin, Jiangxi, China	Hort l6A	Leaf	2024	3	2.6
8	F8	Fengxin, Jiangxi, China	Hort l6A	Trunk	2024	3	2.6
9	G9	Gaoan, Jiangxi, China	Hongyang	Leaf	2024	3	2.4
10	G10	Gaoan, Jiangxi, China	Hongyang	Leaf	2024	3	2.4
11	G11	Gaoan, Jiangxi, China	Hongyang	Trunk	2024	3	2.4
12	G12	Gaoan, Jiangxi, China	Hongyang	Trunk	2024	3	2.4
13	G13	Gaoan, Jiangxi, China	Hort l6A	Branch	2024	3	2.6
14	G14	Gaoan, Jiangxi, China	Hort l6A	Branch	2024	3	2.0
15	J15	Jingdezhen, Jiangxi, China	Hongyang	Trunk	2023	3	2.2
16	J16	Jingdezhen, Jiangxi, China	Hongyang	Branch	2023	3	2.4
17	J17	Jingdezhen, Jiangxi, China	Hongyang	Trunk	2023	3	2.4
18	J18	Jingdezhen, Jiangxi, China	Hongyang	Branch	2023	3	2.4
19	J19	Jingdezhen, Jiangxi, China	Hongyang	Leaf	2023	3	2.4
20	J20	Jingdezhen, Jiangxi, China	Hongyang	Leaf	2023	3	2.6
21	J21	Jingdezhen, Jiangxi, China	Hongyang	Leaf	2023	3	2.2
22	W22	Wuning, Jiangxi, China	Hort l6A	Branch	2024	3	1.8
23	W23	Wuning, Jiangxi, China	Hongyang	Branch	2024	3	1.8
24	W24	Wuning, Jiangxi, China	Hort l6A	Branch	2024	3	2.2
25	W25	Wuning, Jiangxi, China	Hort l6A	Leaf	2024	3	2.0
26	W26	Wuning, Jiangxi, China	Hongyang	Leaf	2024	3	2.2
27	W27	Wuning, Jiangxi, China	Hongyang	Leaf	2024	3	2.4
28	S28	Shangrao, Jiangxi, China	Hongyang	Leaf	2024	3	2.4
29	S29	Shangrao, Jiangxi, China	Hongyang	Leaf	2024	3	2.4
30	S30	Shangrao, Jiangxi, China	Hongyang	Branch	2024	3	2.6
31	S31	Shangrao, Jiangxi, China	Hongyang	Leaf	2024	3	2.6
32	S32	Shangrao, Jiangxi, China	Hongyang	Branch	2024	3	2.6
33	S33	Shangrao, Jiangxi, China	Hongyang	Branch	2024	3	2.6
34	S34	Shangrao, Jiangxi, China	Hongyang	Trunk	2024	3	2.2
35	X35	Xunwu, Jiangxi, China	Hongyang	Branch	2024	3	2.4
36	X36	Xunwu, Jiangxi, China	Hongyang	Branch	2024	3	2.4
37	X37	Xunwu, Jiangxi, China	Hongyang	Branch	2024	3	2.4
38	X38	Xunwu, Jiangxi, China	Hort l6A	Leaf	2024	3	2.4
39	X39	Xunwu, Jiangxi, China	Hort l6A	Leaf	2024	3	2.2
40	X40	Xunwu, Jiangxi, China	Hort l6A	Leaf	2024	3	2.2
41	X41	Xunwu, Jiangxi, China	Hongyang	Leaf	2024	3	2.2
42	X42	Xunwu, Jiangxi, China	Hongyang	Branch	2024	3	2.4

### 2.2 Pathogenicity tests

According to the method of (Hoyte et al., 2015) and (Qin et al., 2023), pathogenicity tests of all Psa isolates were performed on the soft green stems *in vitro* from healthy *A. deliciosa* cv. Hongyang. The healthy stems (10–15 cm long) in vitro were sterilized and then wounded, injected with 20 uL of Psa bacterial suspension (10^8^ CFU/mL) using a sterile syringe. Similarly, sterile saline solution served as a negative control. Each treatment was repeated thrice. Stems were placed in a constant temperature incubator at 20 °C, with 12 h of light and 12 h of darkness for 15 days. Fifteen days after inoculation, the pathogenesis of the materials was observed. Finally, bacterial colonies reisolated from the disease symptoms of Hongyang green branches inoculated with Psa were identified by PCR tests using Psa-specific primer PsaF1/PsaR2 (Rees-George et al., 2010).

### 2.3 DNA extraction and PCR amplification

Bacterial isolates were cultured overnight in 5 mL of liquid LB medium at 28 °C and shaken at 200 rpm. Total genomic DNA was extracted using Tianamp Bacteria DNA Kit (Tiangen biochemical technology (Beijing) Co.; Ltd.) and stored at −20 °C.

Molecular confirmation was by PCR amplification of two sets of Psa-specific primers KNF/R, AvrDdpx-F/R and PSAF1/R2, yielding 492 bp, 226 bp, and 280 bp amplicons, respectively, were used for the molecular characterization of 42 isolates (Table 1). Duplex PCR with the primers KNF/R and AvrDdpx, Psa-specific primer pair PsaF1/PsaR2 were performed following the programs using a previously described method (Koh and Nou, 2002; Rees-George et al., 2010). DNA of the Psa strain M228 (CP032631, biovar 3, Shaanxi, China) was used as a positive control, and nuclease-free water was used as a negative control in the PCR tests. PCR products were detected by 1% agarose gel electrophoresis, and then sent to Shanghai Bioengineering Co., Ltd. for sequencing. The sequences were compared with those of standard strains in the GenBank database by BLAST to determine their taxonomic status.

For further Psa biovar analysis, specific sequences were obtained by PCR amplification with the all 42 Psa extracted DNA as a template and Psa biovar-specific primers PsaJ-F/PsaJ-R (biovar 1) (Lee et al., 2016), PsaK-F/PsaK-R (biovar 2) (Lee et al., 2016), hopZ5-F2/hopZ5-R2 (biovar 3) (Pei et al., 2023), hopO1-F/hopO1-R (biovar 5) (Fujikawa and Sawada, 2016) and Con044F/Con044R (biovar 6) (Fujikawa and Sawada, 2019). DNA of the Psa strains M228 was used as a positive control, and H_2_O was used as a negative control in the PCR tests. Primers and annealing temperatures used in the PCRs are listed in Supplementary Table S1. Each PCR assay was repeated three times.

### 2.4 Phylogenetic analysis by MLSA

To identify the biovar of the *P. syringae* pv. *actinidiae* isolates from different geographical regions in Jiangxi Province, multilocus sequence typing was carried out by amplifying the six housekeeping genes *acnB* (Aconitate hydratase B), *cts* (Citrate synthase), *gapA* (glyceraldehyde-3-phosphate dehydrogenase), *gyrB* (DNA gyrase B), *pfk* (Phosphofructokinase), and *rpoD* (sigma factor 70). All 42 isolates (Table 1) were identified using the primers listed in Supplementary Table S1 as previously described (Sarkar and Guttman, 2004). PCR products were detected by agarose gel electrophoresis, and then sent to Shanghai Bioengineering Co., Ltd. for sequencing. A nucleotide BLAST search was performed to obtain the six housekeeping gene sequences against sequences of biovars 1 to 6 of Psa available in GenBank. The nucleotide sequences of the *acnB*, *cts*, *gapA*, *gyrB*, *pfk*, and *rpoD* genes of other Psa biovars available in GenBank were included in the analysis and are listed in Supplementary Table S2. The sequences of each locus were aligned using the CLUSTALW included in the MEGA7 software. A dendrogram from four-locus concatenated sequences was generated using neighbor-joining (UPGMA) and 1,000 bootstrap iterations.

### 2.5 Chemical agent sensitivity test

The fungicide was diluted to concentrations of 50, 100, 200, 400, and 600-fold to establish five gradient dilutions. The inhibitory zone diameters for each concentration were determined using the K-B paper method, following the previously described procedure (Zhu et al., 2023; Liu et al., 2025). Briefly, the representative strain F7 was streaked onto an LB plate and activated at 28 °C. A single colony was then inoculated into 5 mL of sterilized LB broth and cultured at 28 °C with shaking at 200 rpm for 24 h. The resulting bacterial suspension was diluted with sterile water to an OD_600_ of 0.5. One milliliter of this suspension was added to 100 mL of molten LB agar (approximately 45 °C), mixed thoroughly, and poured into Petri dishes to prepare the plates. Sterilized and dried neutral filter paper discs (5 mm in diameter) were placed on the agar surface, with one disc per plate. Each disc was impregnated with 10 μL of fungicide solution at a specific concentration. Six replicates were performed for each concentration. Sterile water was used as a blank control. The plates were incubated at 28 °C, and after 72 h, the zones of inhibition were observed and photographed. The diameters of the inhibition zones were measured using the cross-method, and the inhibition rates were calculated accordingly. The EC_50_ values represent the concentrations required to achieve 50% efficacy, calculated using toxicity regression analysis with DPS software (version 7.05). The EC_50_ value was determined based on three independent replicate experiments.

### 2.6 Copper resistance assays

Copper resistance was evaluated by determining the minimal concentration of copper that inhibited growth (minimal inhibitory concentration, MIC) on mannitol-glutamate yeast extract medium (MGY) plates supplemented with CuSO_4_·5H_2_O at concentrations of 0, 0.30, 0.60, 0.80, 1.0, 1.2, 1.4, 1.6, 1.8, 2.0, 2.2, 2.4, 2.6, and 3.20 mM for the sensitivity assays (Bender and Cooksey, 1986; Horuz et al., 2025). Psa strains were considered resistant when their MIC exceeded 0.8 mM CuSO_4_·5H_2_O, based on Cazorla’s criteria (Aiello et al., 2015).

## 3 Results

### 3.1 Description of disease symptoms

**Figure 1:**
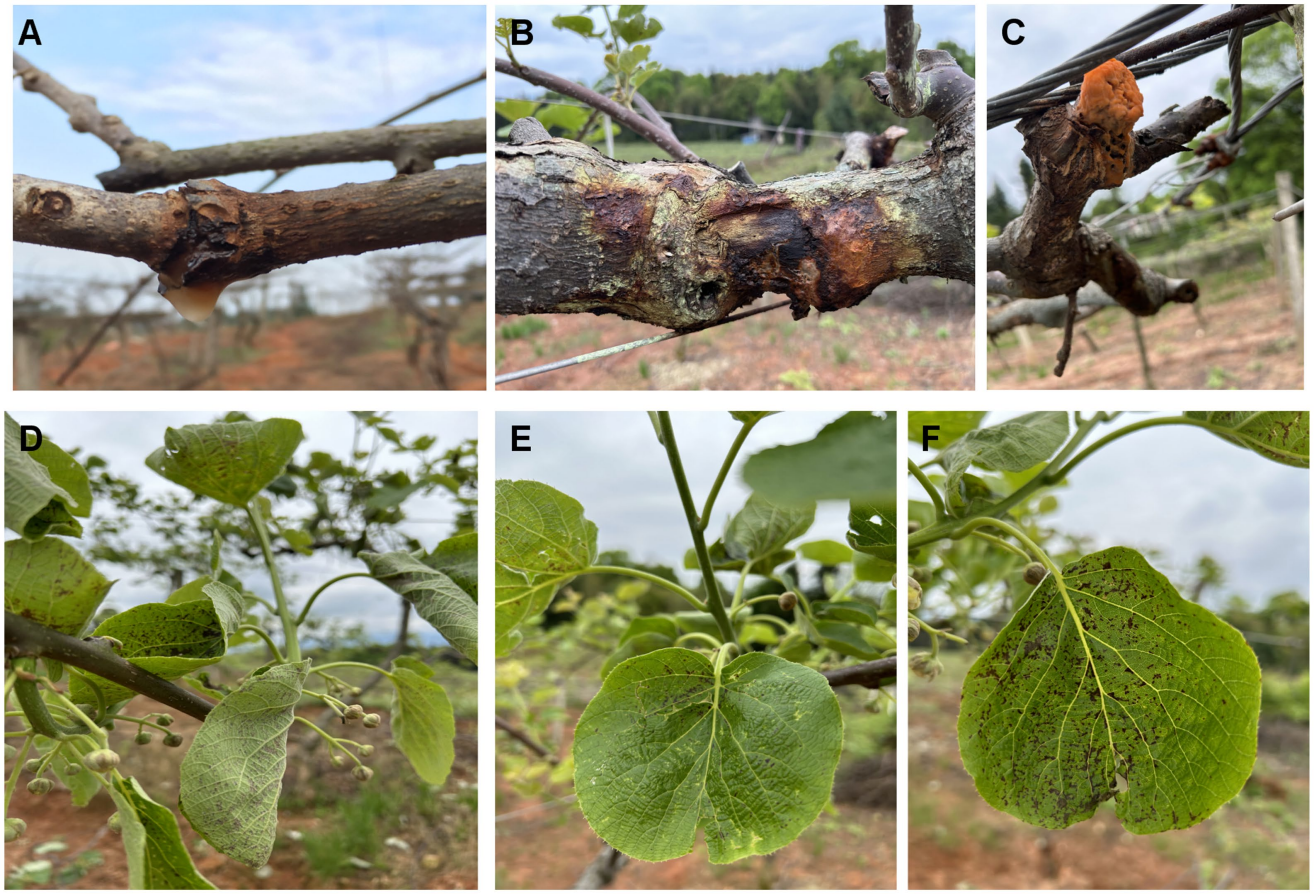
Kiwifruit bacterial canker symptoms. **(A–C)** Dark-red bacterial ooze running down on the trunk. **(D–F)** Small, dark brown spots surrounded by yellow halos on upper leaf surface.

Typical symptoms of bacterial canker were observed in kiwifruit orchards across six major production areas in Jiangxi Province (Figure 1). The disease onset usually began in February or early March, characterized by raised, cracked lesions on trunks and branches that exuded a typical milky bacterial ooze. This exudate later turned yellowish-brown to reddish-brown. Adjacent to these lesions, reddish-brown necrotic spots were often present. Ultimately, infected shoots withered and exhibited dieback symptoms (Figures 1A–C). By early May, characteristic leaf symptoms appeared, presenting as brown, irregular, or angular necrotic spots frequently surrounded by a yellow halo (Figures 1D–F). In some orchards, symptoms including shoot blight and bacterial decay persisted even until mid-July. A total of 183 symptomatic samples of leaves, shoots, and trunks were collected from these six regions in Jiangxi Province, China.

### 3.2 Bacterial isolation and phenotypic characterization

**Figure 2:**
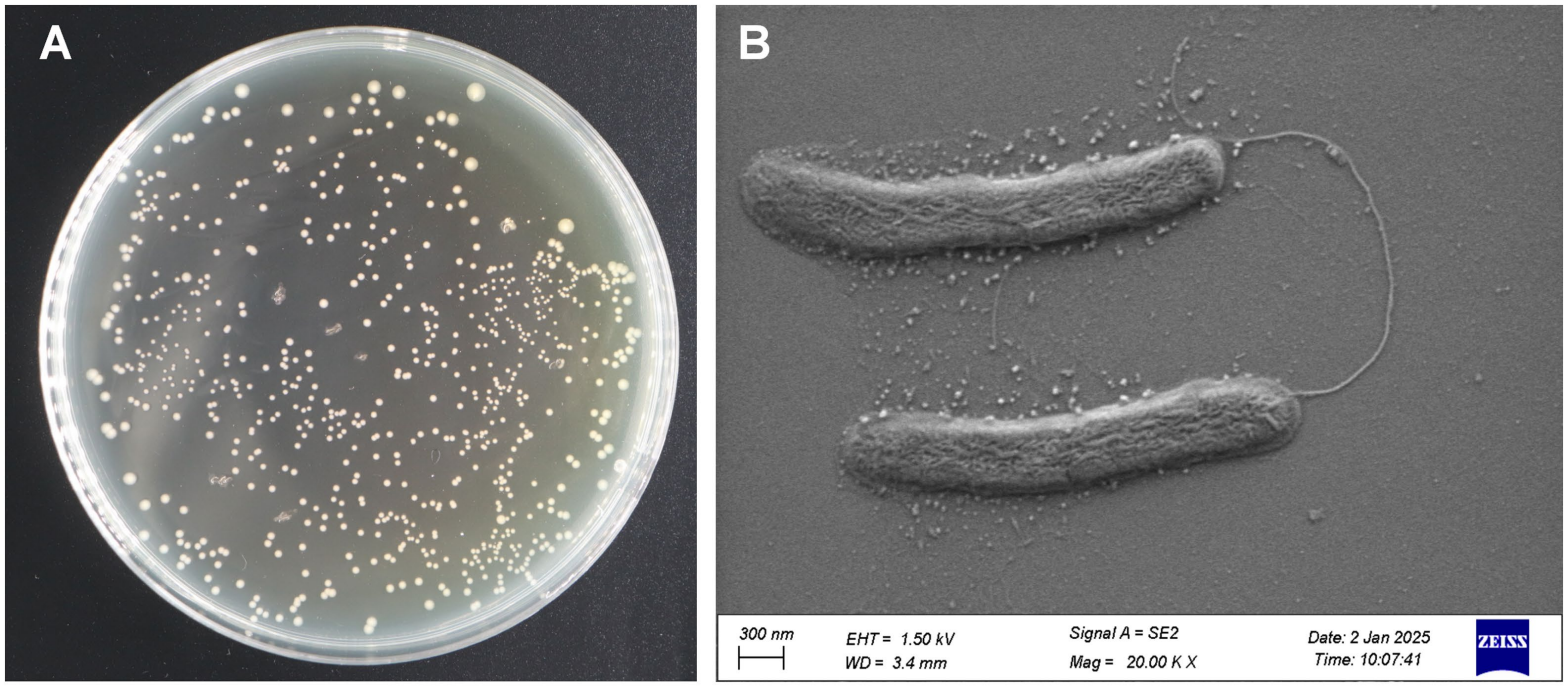
Colony morphology and electron microscopy of *Pseudomonas syringae* pv. *actinidiae*. **(A)** Bacterial colonies grown on NA medium for 48 h at 28 °C. **(B)** Electron microscopy showing cell morphology and flagella.

From the collected diseased samples, 42 bacterial strains were isolated and purified (Table 1). All presumptive Psa isolates formed round, creamy white, mucoid, convex, and smooth colonies on NA medium. Growth was relatively slow; after 24 h of incubation, colony diameters ranged from 1 to 2 mm (Figure 2A). All strains obtained in this study were Gram-negative. Electron microscopy revealed that the representative strain F7 was rod-shaped, with blunt round ends and flagella (Figure 2B). The colony morphology was consistent with previous descriptions for *Pseudomonas syringae* pv. *actinidiae* (Pei et al., 2023; Qin et al., 2023).

### 3.3 Molecular identification of isolates

All 42 isolates were confirmed as *Pseudomonas syringae* pv. *actinidiae* (Psa) through molecular assays. Duplex PCR using primer pairs KNF/R and AvrDdpx-F/R, which amplify 492 bp and 226 bp fragments, respectively, yielded the expected amplicons (Supplementary Figure S1). Furthermore, PCR with the PSAF1/R2 primer pair, specific for Psa, successfully amplified a 280 bp fragment from all isolates (Supplementary Figure S2).

**Figure 3:**
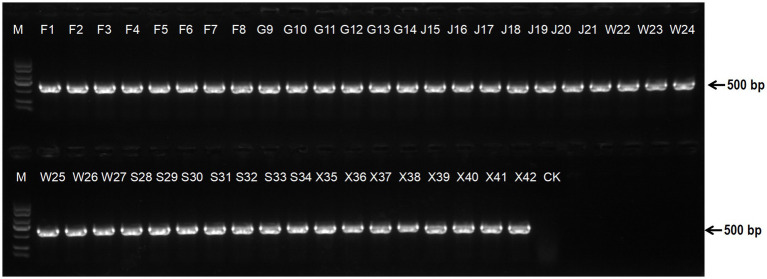
Identification of a biovar of *Pseudomonas syringae* pv. *actinidiae* isolates based on Psa biovar3-specific primer HopZ5-F/HopZ5-R, designed to amplify a 500 bp fragment.

Subsequently, biovar-specific PCR was performed using five primer sets (biovar 1–5). A specific amplicon corresponding to the *hopZ5* gene, a marker for Psa biovar 3, was successfully obtained from all isolates (Figure 3). The other four pairs of biovar-specific primers cannot amplify specific bands (data not shown for brevity). These results indicate that all 42 strains isolated from Jiangxi Province belong to *P. syringae* pv. *actinidiae* biovar 3.

### 3.4 Pathogenicity assays

**Figure 4:**
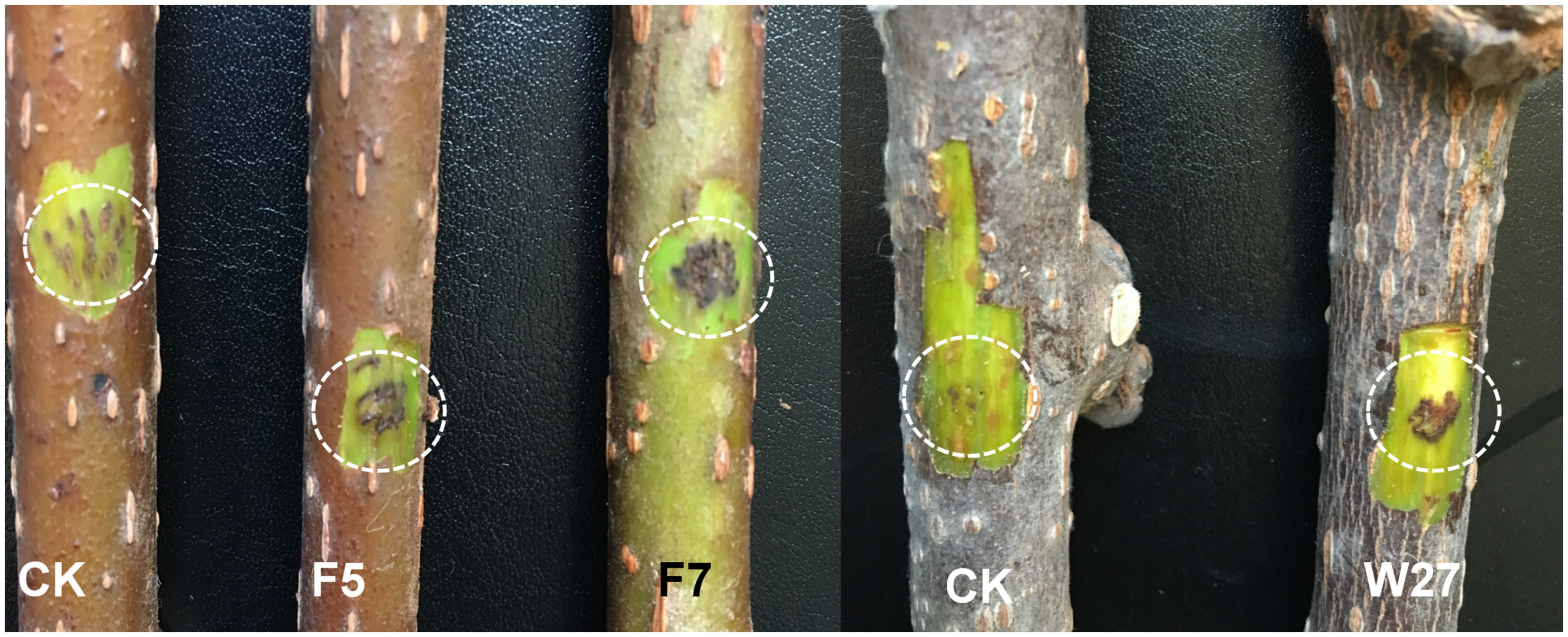
Disease symptoms on branches of kiwifruit at 15 days after inoculation with bacterial suspension of *Pseudomonas syringae* pv. *actinidiae* strains (F5, F7, W27). CK denotes the control.

All tested strains were confirmed to be pathogenic on detached kiwifruit stems. Within 15 days post-inoculation, all Psa isolates caused disease in the kiwifruit cultivar ‘Hongyang’, resulting in browning of the local phloem and xylem and the production of bacterial exudate in necrotic tissues. No disease symptoms developed on the control stems (Figure 4; Supplementary Figure S3). These symptoms were similar to those caused by natural infections. Bacteria were re-isolated from the margin of these characteristic lesions and identified as Psa using Psa-specific primer (PSAF1/R2), thereby fulfilling Koch’s postulates.

### 3.5 Phylogenetic analysis by MLSA

**Figure 5:**
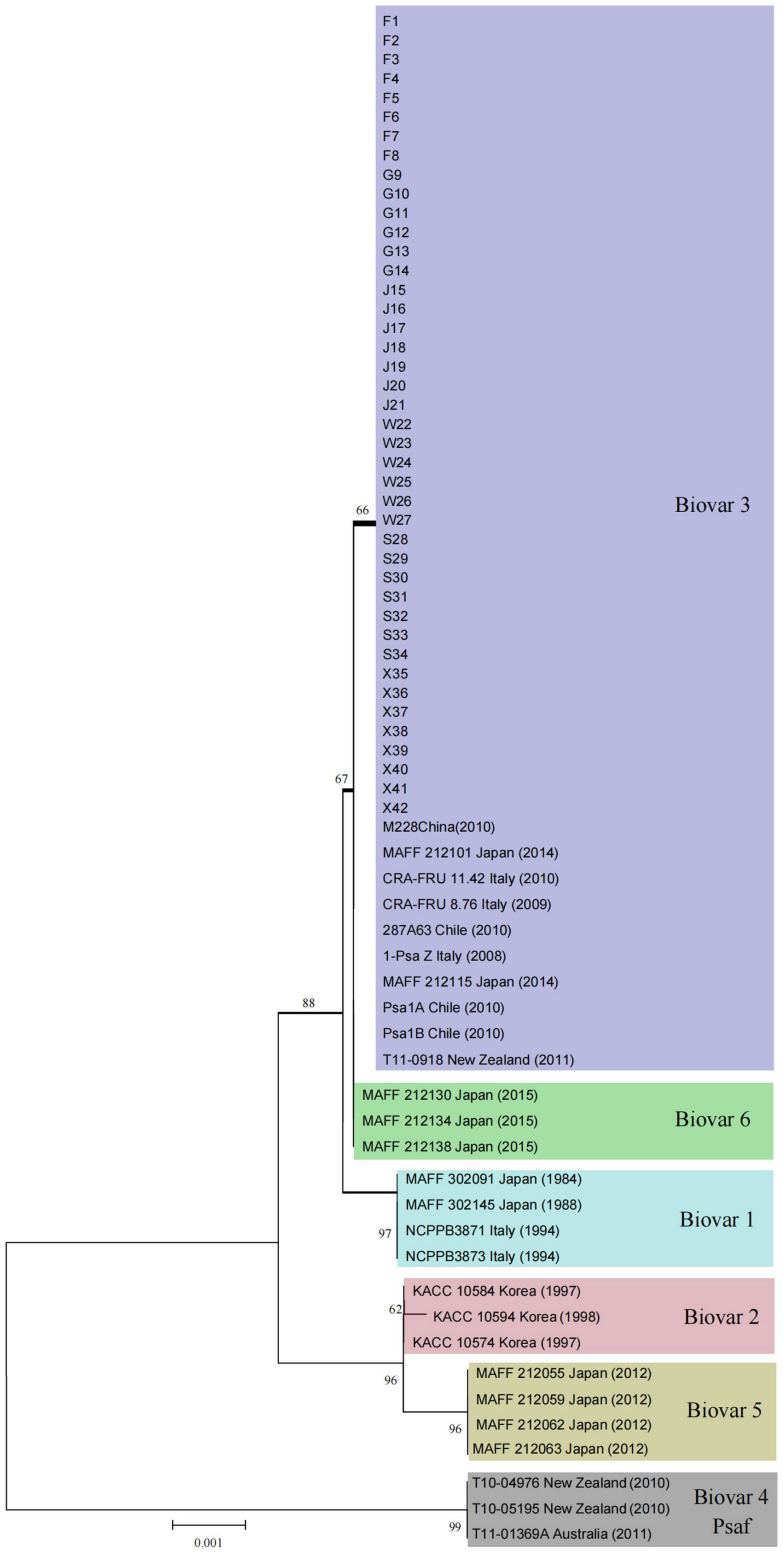
Phylogenetic tree of *Pseudomonas syringae* pv. *actinidiae* isolates derived from multilocus sequence analysis (MLSA). Phylogenetic tree using the neighbor-joining method (bootstrap: 1,000 replicates) and concatenated sequences of the genes *acnB*, *cts*, *gapA*, *gyrB*, *pfk*, and *rpoD* for each isolate. Country, year of isolation, and biovar clade are indicated for each strain.

MLSA analysis using the six housekeeping genes *acnB*, *cts*, *gapA*, *gyrB*, *pfk*, and *rpoD* allowed the comparison of the Psa isolates from Jiangxi Province with reference strains of Psa biovar 1–6. The size of the sequence fragment of *acnB*, *cts*, *gapA*, *gyrB*, *pfk*, and *rpoD* genes was 626, 573, 716, 664, 818 and 582 bp, respectively. A Maximum Likelihood phylogenetic tree consisted of 42 Psa isolates from Jiangxi Province was constructed based on the 3,979 bp long concatenated sequence of the six genes, and showed that the genes sequenced have 100% identity with the corresponding genes in different Psa strains belonging to biovar 3, including strains M228 obtained in 2010 in shaanxi, China. Representative sequences of the *acnB*, *cts*, *gapA*, *gyrB*, *pfk*, and *rpoD* genes from isolate F7 have been deposited in GenBank under accession numbers PZ096153-PZ096158. No polymorphisms were found among the 42 concatenated sequences of Jiangxi isolates used for MLSA, nor with the strain M228. Reference and tested isolate sequences were used to construct a phylogenetic tree, showing that all 42 *P. syringae* pv. *actinidiae* test isolates clustered with 10 *P. syringae* pv. *actinidiae* biovar 3 reference strains in all phylogenetic trees (Figure 5).

### 3.6 Chemical agent assays

Building on the above findings, which identified the prevalent and highly pathogenic biovar 3 as the causal agent of bacterial canker in Jiangxi Province, China, we proceeded to evaluate the efficacy of 10 chemical agents (as listed in Supplementary Table S3) against the representative strain F7. In a preliminary screening, except for basic copper sulfate, all other test reagents inhibited the growth of the representative strain F7. Three agents-tetramycin, bronopol, and ethylicin-demonstrated particularly strong inhibitory effects and were selected for further dose–response assays to determine their half-maximal effective concentrations (EC_50_). As summarized in Table 2, tetramycin was the most potent, with an EC_50_ value of approximately 0.002 mg/L, followed by bronopol (approx. 0.068 mg/L) and ethylicin (approx. 7.104 mg/L). These results indicate that, under the tested conditions, tetramycin is the most effective chemical agent against the prevalent Psa strain in Jiangxi.

**Table 2:** Inhibitory effects of various chemical agents on strain F7.

Bactericides name	Toxic regression equation	EC_50_ (mg/L)	Correlation coefficent
Tetramycin 0.3%, AS	y = 0.436x + 1.229	0.002	0.971
Bronopol 20%, WP	y = 0.437x + 0.511	0.068	0.984
Ethylicin 80%, EC	y = 0.999x - 0.850	7.104	0.963
Benzisothiazolinone 1.5%, EW	y = 1.44x - 1.873	19.974	0.956
Zhongshengmycin 3%, WP	y = 1.001x - 1.558	36.069	0.955
Kasugamycin 2%, AS	y = 3.43x - 7.505	154.202	0.935
Cuaminosulfate 15%, AS	y = 1.541x - 4.245	568.609	0.972
Chloroisobromine cyanuric acid 50%, SP	y = 1.767x - 5.199	874.611	0.991
Copper Hydroxide 46.1%, WG	y = 1.612X - 5.211	1705.242	0.980
Basic copper sulfate 30%, SC	–	–	–

SC, suspension concentrate; SP, Soluble Powder; WG, water-dispersible granule; EW, Emulsion, oil in water; EC, emulsifiable concentrates; WP, wettable powder; AS, aqueous solutions.

### 3.7 Investigations of copper resistance

Copper-based compounds are among the most commonly used agents for controlling Psa. However, their extensive application has led to the emergence of copper-resistant Psa strains (Colombi et al., 2017; Cesari et al., 2023). Consistent with this trend, our study found that the representative strain F7 exhibited low sensitivity to copper-based bactericides (Table 1). Furthermore, *in vitro* copper resistance assays conducted on all 42 Psa strains revealed that every isolate was capable of growth at a concentration of ≥1.8 mM CuSO₄·5H₂O, with no copper sulfate-sensitive strains detected. This minimum inhibitory concentration far exceeds the standard resistance threshold of 0.8 mM. These results suggest a widespread and potentially high level of copper resistance within the Psa populations sampled in Jiangxi Province, highlighting a significant risk to current control strategies.

## 4 Discussion

Bacterial canker of kiwifruit, caused by *Pseudomonas syringae* pv. *actinidiae* (Psa), poses a significant and ongoing constraint to the global kiwifruit industry (He et al., 2019; da Nunes Silva et al., 2022). The persistence and management challenges of this disease are influenced by the variability within the pathogen population, as different strains exhibit varying degrees of pathogenicity on their hosts (Zhao et al., 2019). Consequently, the detection and characterization of local Psa populations are critical for developing effective, region-specific prevention and control strategies.

In this study, we isolated and characterized Psa strains from Jiangxi Province, a major kiwifruit-growing region in southeastern China. All 42 obtained strains were identified as Psa biovar 3 through morphological assessment, duplex PCR, and multilocus sequence analysis (MLSA). This finding aligns with previous reports from other Chinese provinces, such as Shaanxi and Sichuan, where only biovar 3 has been identified to date (He et al., 2019; Pei et al., 2023). These results also suggest that no new biovars have been introduced to Jiangxi Province, nor the wider region of China, during this period. While China is the center of origin for Actinidia species, the exclusive presence of a single biovar is notable. It contrasts with the higher biovar diversity observed in countries such as Japan (biovars 1, 3, 4, 5, and 6) (Poulter et al., 2017), Korea (biovars 1, 2, and 3) (Kim et al., 2017), and Italy (biovars 1 and 3) (Cesari et al., 2023). This global distribution pattern strongly implies that the international trade of kiwifruit plant material has been a primary driver for the transboundary introduction of different Psa biotypes. The high diversity in Japan, for instance, may reflect a history of extensive germplasm exchange. The absence of biovars 1, 2, and others in China, despite being the genetic origin of the host, raises intriguing questions. It suggests that either these biovars have not been introduced, or they have been introduced but failed to establish due to competitive exclusion by the well-adapted biovar 3, different environmental conditions, or host resistance profiles. Continuous monitoring using advanced molecular tools is therefore imperative to detect any future incursions of new biovars, especially given the ongoing global plant trade.

Pathogenic variation among Psa biovars is linked to differences in phytotoxin production, a key virulence factor (Poulter et al., 2017; Zhao et al., 2019; Fujikawa et al., 2020). Biovar 1 produces phaseolotoxin, biovar 2 produces coronatine, biovars 3, 4, and 5 produce neither, while biovar 6 can produce either toxin (Fujikawa and Sawada, 2019). The predominance of the non-toxigenic biovar 3 in China warrants further investigation and continued monitoring. The lack of these toxin genes in biovar 3 suggests that its high virulence, which has caused pandemics since 2008, must be driven by other virulence factors, such as the abundant Type III Secretion System (T3SS) effectors (Hu et al., 2025). This highlights the complexity of the Psa-kiwifruit pathosystem and warrants further genomic and transcriptomic studies to understand the molecular basis of biovar 3’s global success in the absence of classical phytotoxins.

The management of bacterial canker remains difficult. The limited number of effective treatments, combined with environmental concerns over copper-based compounds and emerging antibiotic resistance, underscores the need for environmentally sound control strategies (Colombi et al., 2017; Cesari et al., 2023). Our *in vitro* assays identified three agents (tetramycin, bronopol, and ethylicin) with strong inhibitory effects against Psa strain F7. Tetramycin was particularly potent. However, it should be noted that the chemical sensitivity tests were performed on only one representative strain (F7); due to the possible genetic and phenotypic diversity among strains from different regions (as shown in Figure 4), other isolates may exhibit different sensitivities to the tested agents. Further studies with a broader panel of isolates are needed to confirm these findings. Nevertheless, these findings are consistent with the growing body of research exploring alternatives to conventional copper-based treatments (Wang et al., 2021; Wang et al., 2025). In China, agricultural extension services have recommended antibiotics such as tetramycin among the options for bacterial canker control during key phenological periods. Bronopol and ethylicin represent additional chemical classes with distinct modes of action that could prove valuable in resistance management strategies. These compounds represent promising candidates for further field efficacy trials. To mitigate resistance development, future disease management should consider the rotation or combination of these agents.

A critical and concerning finding of this study is the widespread and high-level copper resistance among all Psa isolates from Jiangxi Province. All tested strains grew at copper concentrations (MICs 1.80–2.60 mM) far exceeding the conventional resistance threshold of 0.8 mM. This phenomenon is not isolated to Jiangxi Province, China; it mirrors emerging reports of copper-resistant Psa strains from Europe, New Zealand, and Japan, where intensive copper use has exerted strong selective pressure on pathogen populations (Aiello et al., 2015; Colombi et al., 2017; Cesari et al., 2023). The variation in MIC levels reported globally highlights how local agricultural practices and pathogen adaptation can shape resistance dynamics.

These results collectively emphasize the urgent need for integrated disease management strategies that reduce reliance on copper. Long-term monitoring of resistance trends is essential for sustainable kiwifruit cultivation. Future research should focus on discovering novel compounds with different modes of action, elucidating the molecular mechanisms of copper resistance, and developing region-specific, sustainable management protocols. The findings from this study provide a foundation for refining such strategies against Psa in China.

## 5 Conclusion

This work confirms the prevalence of the aggressive Psa biovar 3 across Jiangxi’s kiwifruit areas and reveals widespread, high-level copper resistance in local strains. The significant efficacy of alternative agents, especially tetramycin, provides practical candidates for field evaluation. Sustainable control of bacterial canker in the region must therefore shift from copper-dependent programs toward integrated approaches that combine rotating effective compounds, continuous pathogen monitoring, and resistant-variety deployment. These findings establish a critical foundation for science-based disease management aimed at securing the long-term productivity of kiwifruit orchards in southeastern China.

## Data Availability

The datasets presented in this study can be found in online repositories. The names of the repository/repositories and accession number(s) can be found in the article/Supplementary material.
