# Supplementary material for: Prevalence of copper-resistant Pseudomonas syringae pv. actinidiae biovar 3 in Jiangxi Province, China
Source: Front Microbiol. 2026 Apr 29;17:1824303. doi: 10.3389/fmicb.2026.1824303 (PMC13167956; doi:10.3389/fmicb.2026.1824303)
Supplement: Supplementary file 1 [file Table_1.docx]

Supplementary Material

# Supplementary Figures


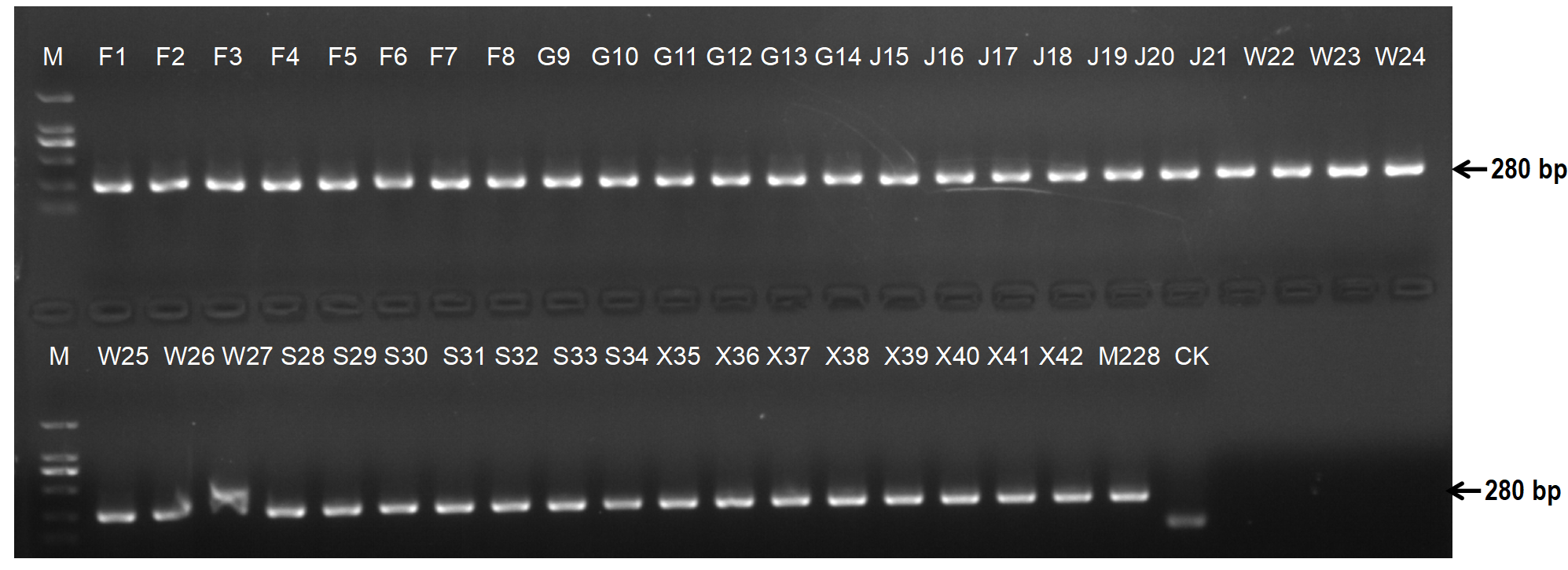


**Supplementary Figure 1.** Identification of *Pseudomonas syringae* pv. *actinidiae* isolates based on duplex-PCR. Duplex PCR using Psa-specific primer pairs KNF/R and AvrDdpx-F/R, which amplify 492 bp and 226 bp fragments, respectively.


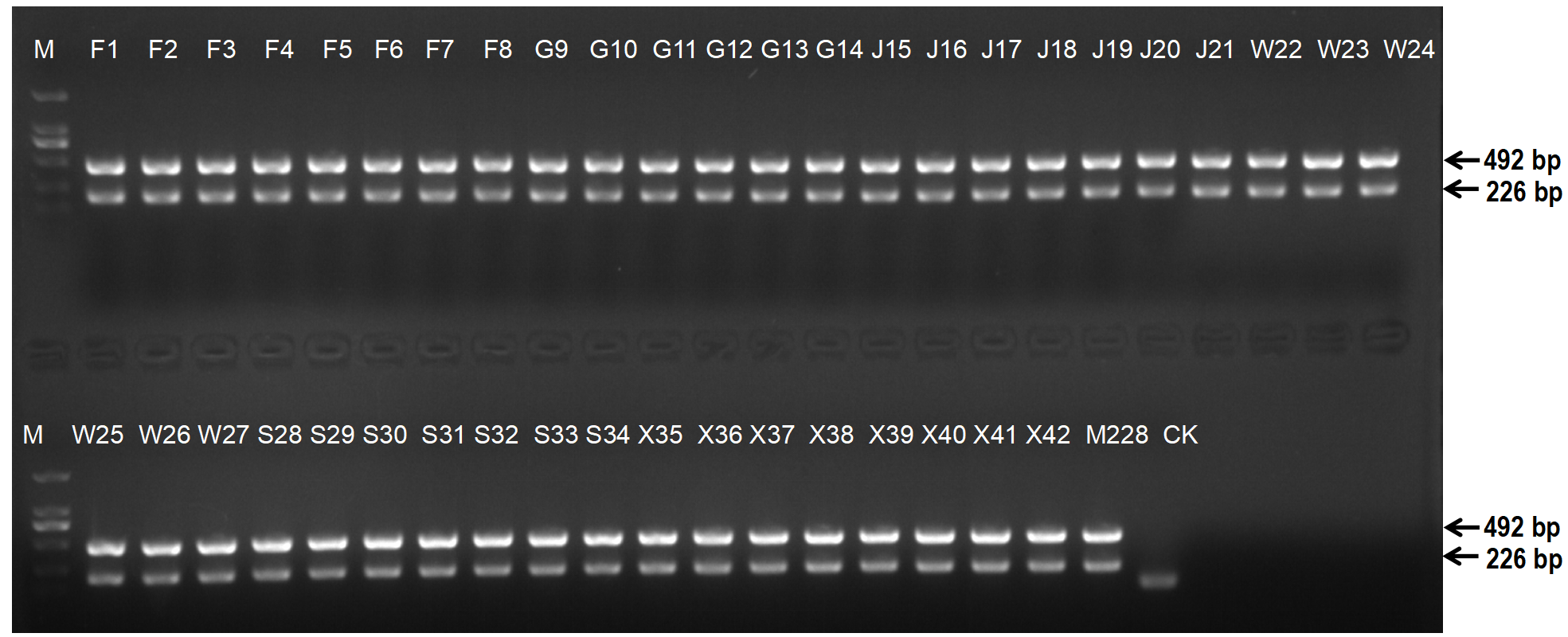


**Supplementary Figure 2.** Identification of *Pseudomonas syringae* pv. *actinidiae* isolates based on PCR. PCR analysis using Psa-specific primer pair PSA-F/PSA-R, designed to amplify a 280-bpfragment.

**
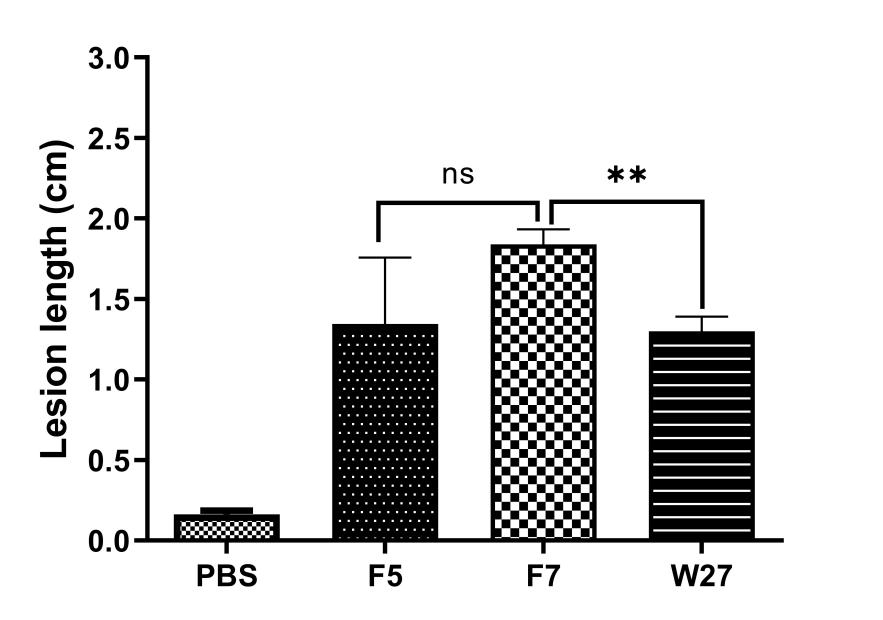
Supplementary Figure 3.** Lesion lengths on branches of kiwifruit at 15 days after inoculation with bacterial suspension of *Pseudomonas syringae* pv. *actinidiae* strains (F5, F7, W27). PBS denotes the control (CK). The asterisks above the error bars indicate significant differences compared with the F7 strain (***P* < 0.01). All experiments were repeated three times with similar results.

# Supplementary Tables

**Tables S1*.* Primer sequences for molecular identification of *Pseudomonas syringae* pv. *actinidiae.***

| Primer name | Primer sequence (5’) | Amplicon size (bp) | Reference |
| --- | --- | --- | --- |
| PSAF1 | TTTTGCTTTGCACACCCGATTTT | 280 | Rees-George et al., 2010 |
| PSAR2 | CACGCACCCTTCAATCAGGATG | - |  |
| AvrDdpx-F | TTTCGGTGGTAACGTTGGCA | 226 | Koh and Nou, 2002 |
| AvrDdpx-R | TTCCGCTAGGTGAAAAATGGG | - |  |
| KNF | CACGATACATGGGCTTATGC | 492 | Koh and Nou, 2002 |
| KNR | CTTTTCATCCACACACTCCG | - |  |
| PsaJ-F | GACGTCGACGACAAGGTGAT | 481 | Lee et al., 2016 |
| PsaJ-R | AGTAAACCGTGCCGTCATCTC | - |  |
| PsaK-F | GACAAAGCCAAAAAGGCGA | 413 | Lee et al., 2016 |
| PsaK-R | AGTAAACCGTGCCGTCATCTC | - |  |
| hopZ5-F2 | CAGGAATTCATGACTTCTCA | 665 | Pei et al., 2023 |
| hopZ5-R2 | TAGTCTCGAAGATTCAATGG | - |  |
| hopO1-F | TCAGCTCAATCCACAGCATC | 854 | Fujikawa and Sawada, 2016 |
| hopO1-R | TACCCGGTTTGAAACGACTC | - |  |
| Con044F | AAGCGCCTTAATCTCGTTCA | 470 | Fujikawa and Sawada, 2019 |
| Con044R | ATTCCGGATTGGGTATCACA | - |  |
| *acn*-F | ACATCCCGCTGCACGCYCTGGCC | 673 | Sarkar and Guttman, 2004 |
| *acn*-R | GTGGTGTCCTGGGAACCGACGGTG | - |  |
| *cts*-F | AGTTGATCATCGAGGGCGCWGCC | 618 | Sarkar and Guttman, 2004 |
| *cts*-R | TGATCGGTTTGATCTCGCACGG | - |  |
| *gapA*-F | CGCCATYCGCAACCCG | 716 | Sarkar and Guttman, 2004 |
| *gapA*-R | CCCAYTCGTTGTCGTACCA | - |  |
| *gyrB*-F | GGGCGGYAAGTTCGAYGACAAYTC | 665 | Sarkar and Guttman, 2004 |
| *gyrB*-R | TAATBGCRGTCARRCCTTCRCGSGC | - |  |
| *pfk*-F | ACCMTGAACCCKGCGCTGGA | 854 | Sarkar and Guttman, 2004 |
| *pfk*-R | ATRCCGAAVCCGAHCTGGGT | - |  |
| *rpoD*-F | AGGCGARATCGAAATCGCCAARCG | 582 | Sarkar and Guttman, 2004 |
| *rpoD*-R | GGAACTGSCGCAGGAAGTCGGCRCG | - |  |

**Table S2. Sequences of Psa Biovars used in MLSA analysis.**

| **Biovar** | **Strain** | **Origin** | **year** | **Accession number NCBI: *acnB* / *cts* / *gapA* / *gyrB*/ *pfk* / *rpoD*** |
| --- | --- | --- | --- | --- |
| 1 | MAFF 302145 | Japan | 1988 | JN683396 / JN683510 / JN683491 / JN683472 / JN683453 / JN683415 |
|  | MAFF 302091 | Japan | 1984 | JN683394 / JN683508 / JN683489 / JN683470 / JN683451 / JN683413 |
|  | NCPPB 3871 | Italy | 1994 | GU058959 / GU058929 / KF937495 / KF937689 / JX288097 / KF937786 |
|  | NCPPB 3873 | Italy | 1994 | JX287929 / JX287995 / JX288012 / JX288029 / JX288096 / JX287912 |
| 2 | KACC 10754 | Korea | 1997 | JN683393 / JN683507 / JN683488 / JN683469 / JN683450 / JN683412 |
|  | KACC 10584 | Korea | 1997 | JN683391 / JN683505 / JN683486 / JN683467 / JN683448 / JN683410 |
|  | KACC 10594 | Korea | 1998 | JN683392 / JN683506 / JN683487 / JN683468 / JN683449 / JN683411 |
| 3 | CRA-FRU 8.76 | Italy | 2009 | JX287932 / JX287998 / JX288015 / JX288032 / JX288099 / JX287915 |
|  | CRA-FRU 11.42 | Italy | 2010 | JX287935 / JX288000 / JX288017 / JX288034 / JX288102 / JX287917 |
|  | MAFF 212101 | Japan | 2014 | LC008151 / LC008157 / LC008163 / LC008169 / LC008175 / LC008187 |
|  | MAFF 212115 | Japan | 2014 | LC008155 / LC008161 / LC008167 / LC008173 / LC008179 / LC008191 |
|  | T11-0918 | New Zealand | 2010 | JN683383 / JN683476 / JN683495 / JN683457 / JN683440 / JN683400 |
|  | Psa1A | Chile | 2010 | JQ836583 /JQ836616 / JQ836620 / JQ836624 / JQ836646 / JQ836579 |
|  | Psa1B | Chile | 2010 | JQ836584 / JQ836617 / JQ836621 / JQ836625 / JQ836647 / JQ836580 |
|  | 287A63 | Chile | 2010 | JX287924 / JX287990 / JX288007 / JX288024 / JX288091 / JX287907 |
|  | M228 | China | 2010 | CP032631 |
|  | F7 | China | 2024 | PZ096153 / PZ096154 / PZ096155 / PZ096156 / PZ096157 / PZ096158 |
|  | 1-Psa Z | Italy | 2008 | JX287937 / JX288003 / JX288020 / JX288037 / JX288104 / JX287920 |
| 4 | T10-04976 | New Zealand | 2010 | JN683384 / JN683498/ JN683479 / JN683460 / JN683441 / JN683403 |
|  | T10-05195 | New Zealand | 2010 | JN683387 / JN683501 / JN683482 / JN683463 / JN683444 / JN683405 |
|  | T11-01369A | Australia | 2011 | JQ836585 / JQ836618 / JQ836622 / JQ836626 / JQ836648 / JQ836581 |
| 5 | MAFF 212055 | Japan | 2012 | AB842186 / AB842198 / AB842210 / AB842222 / AB842234 / AB842258 |
|  | MAFF 212059 | Japan | 2012 | AB842190 / AB842202 / AB842214 / AB842226 / AB842238 / AB842262 |
|  | MAFF 212062 | Japan | 2012 | AB842193 / AB842205 / AB842217 / AB842229 / AB842241 / AB842265 |
|  | MAFF 212063 | Japan | 2012 | AB842194 / AB842206 / AB842218 / AB842230 / AB842242 / AB842266 |
| 6 | MAFF 212130 | Japan | 2015 | LC065043 / LC065049 / LC065055 / LC065061 / LC065067 / LC065079 |
|  | MAFF 212134 | Japan | 2015 | LC065045 / LC065051 / LC065057 / LC065063 / LC065069 / LC065081 |
|  | MAFF 212138 | Japan | 2015 | LC065047 / LC065053 / LC065059 / LC065065 / LC065071 / LC065083 |

**Table S3. Concentrations of substances used for bactericides sensitivity assays and their China pesticide registration numbers (http://www.chinapesticide.org.cn).**

| **Bactericides name** | **Registration number** | **Active ingredient** | **Manufacturer** |
| --- | --- | --- | --- |
| Tetramycin 0.3%, AS | PD20171878 | Tetramycin | Liaoning Wkioc Bioengineering Co., Ltd. |
| Bronopol 20%, WP | PD20086031 | bronopol | Dandong City Pesticicle General Factory |
| Ethylicin 80%, EC | PD20101285 | Ethylicin | Kaifeng Dadi Nonghua Biological Technology Co., Ltd. |
| Benzisothiazolinone 1.5%, EW | PD20086023 | Benzisothiazolinone | Shaanxi Xida Huate Technology Industrial Co., Ltd. |
| Zhongshengmycin 3%, WP | PD20151933 | Zhongshengmycin | Jiangxi Zhengbang Crop Protection Co., Ltd. |
| Kasugamycin 2%, AS | **PD54-87** | Kasugamycin | Hokko Chemical Industry Co., Ltd. |
| Copper Hydroxide 46.1%, WG | **LS20083292** | Copper Hydroxide | Shanghai Branch, Du Pont China Holding Co., Ltd. |
| Cuaminosulfate 15%, AS | **PD20101618** | Cuaminosulfate | Shaanxi Xiannong Biotechnology Co., Ltd. |
| Chloroisobromine cyanuric acid 50%, SP | **PD20095663** | Chloroisobromine cyanuric acid | Nanjing Nannong Pesticide Technology Development Co., Ltd. |
| Basic copper sulfate 30%, SC | **PD20100066** | Copper sulfate basic | Baoding Pesticide Factory |

SC, suspension concentrate; SP, Soluble Powder; WG, water-dispersible granule; EW, Emulsion, oil in water; EC, emulsifiable concentrates; WP, wettable powder; AS, aqueous solutions.

# Statistics Methods

An analysis of variance (ANOVA) was conducted on experimental datasets using GraphPad Prism 8.0. Error bars in the figures represent standard deviations (SD) as specified in the figure legends. All data are presented as the mean ± SD from at least three independent experiments. The significance of treatment effects was assessed using F-values, with a significance level of *P* = 0.05.

# References

Fujikawa, T., and Sawada, H. (2016). Genome analysis of the kiwifruit canker pathogen *Pseudomonas syringae* pv. *actinidiae* biovar 5. *Sci Rep*. 6, 21399. doi: 10.1038/srep21399.

Koh, Y.J., and Nou, I.S. (2002). DNA markers for identification of *Pseudomonas syringae* pv. *actinidiae*. *Mol Cells*. 13(2), 309-314. doi: /10.1016/S1016-8478(23)15038-2.

Lee, Y.S., Kim, G.H., Koh, Y.J., Zhuang, Q., and Jung, J.S. (2016). Development of specific markers for identification of biovars 1 and 2 strains of *Pseudomonas syringae* pv. *actinidiae*. *Plant Pathol J*. 32(2), 162-167. doi: 10.5423/PPJ.NT.10.2015.0224.

Pei, Y., Ma, L., Zheng, X., Yao, K., Fu, X., Chen, H., et al. (2023). Identification and genetic characterization of *Pseudomonas syringae* pv. *actinidiae* from kiwifruit in Sichuan, China. *Plant Dis*. 107(10), 3248-3258. doi: 10.1094/PDIS-01-23-0005-RE.

Rees‐George, J., Vanneste, J., Cornish, D., Pushparajah, I., Yu, J., Templeton, M., et al. (2010). Detection of *Pseudomonas syringae* pv. *actinidiae* using polymerase chain reaction (PCR) primers based on the 16S-23S rDNA intertranscribed spacer region and comparison with PCR primers based on other gene regions. *Plant Pathol*. 59(3), 453-464. doi: 10.1111/j.1365-3059.2010.02259.x.
